# Plugging Teeth

**Published:** 1841-12

**Authors:** 


*Plugging Teeth.*—This is one of the most important, and at the same time, one of the most difficult operations in dental surgery; and it is an operation, in the performance of which, comparatively, few have attained to a very high state of excellence, and knowing this, it is to us a matter of surprise that it is so frequently entrusted to individuals of questionable skill. The operation unless well performed, is an injury instead of a benefit to the teeth, and it is as essential that the material employed for filling, be such as will resist the action of the agents to which it has necessarily to be exposed, as that the operation be skilfully gone through with. Many articles have been used for filling teeth, but all experienced and skilful practitioners concur in opinion that gold is the only one that can in all cases be depended on, and yet, there are not wanting those, who, through ignorance of the subject, or with a view to deceive, will assert to the public that an amalgum of crude mercury and silver is greatly preferable.

We have been led to these remarks by several cases of imposition of this kind that have recently come to our notice, and for the purpose of showing how easy it is for an individual to be imposed upon by this species of quackery. As an example, we will mention the case of a lady, Mrs. M. of this city, who called upon us a few days since to obtain our professional services. She had several decayed teeth which she wished to have filled, and inquired, if we could do it with *"cement,"* stating at the same time that she had had three teeth filled with it about ten months before, and that she preferred it to any other article, inasmuch as it had remained in them securely ever since. On examining the teeth which had been filled, we found the *"cement,"* as she designated it, though much oxidized, still in them; the decay however was not arrested, but had gone on, notwithstanding the *"cement,"* fillings, and had committed such devastations upon the teeth, that their preservation was rendered almost hopeless.

This is not a single case, we could mention very many others similar to it, but it is sufficient to show that even the remaining of a plug in a tooth is no evidence that the tooth is in a state of preservation. It is an easy matter to fill some cavities in teeth, and especially such as are larger at the bottom than at the opening, so as to prevent the fillings from coming out, but to fill them so as to effectually arrest the further progress of the decay, is quite a different, and by far, more difficult thing. That it can be done, experience has demonstrated beyond all question or doubt, but so far as our own observations extend upon the subject, and we have taken some pains to inform ourself concerning it, dental caries can seldom if ever be perfectly arrested with any of the *metallic* or *tero metallic "cements,"* that have as yet been employed. We have seen many teeth that were filled with one or other of these preparations, but do not recollect to have ever met with one that had been thus treated, in which the decay was not still going on, and the gum around it, not in an obviously unhealthy condition.

The employment of this description of articles for filling teeth, we are induced to believe, from what we can learn, has been more general in England than in this country, and the injury that has there been produced by it, has been so great, that it has become necessary to denounce the practice in the public newspapers; and in consequence of which, many from the hordes of dental charlatans that have there for the last five or six years, been practising upon the credulity of the unsuspecting, have been compelled to seek other fields for the exercise of their vile imposition. New York, we understand, has recently been honoured with a visit from one of these notorious impostors, who recently figured very conspicuously in a London police office.

But, this is an inventive age, and among the inventions by which it has been characterized, those for deceiving, have fully kept pace with those that are useful and beneficial. The world seems so fond of novelties, that it is only necessary for a person to proclaim a new discovery, to find enough ready to test its value, no matter at what risk or expense, and though they be deceived a dozen times, appear equally ready to be duped again. The hundreds, and we might say thousands, who flocked to the celebrated *Williams*, the *oculist*, and to the *Crawcours*, the *dentists*, and to many other similar impostors, though proclaimed to be such by those who were competent to judge, furnish incontestible evidence of the truth of this. But experience is said to be a good teacher, and we are getting so much of it, that we shall doubtless ere long become more wise.—*Balt. Ed.*

